# Radial extracorporeal shock wave therapy modulates hamstring stiffness and flexibility in young males with hamstring tightness: a randomized controlled trial

**DOI:** 10.3389/fphys.2026.1927662

**Published:** 2026-09-07

**Authors:** Meixia Zou, Siqi Huang, Kaiyao Hou, Yiming Chen, Peifeng Shen, Wenjie Guo, Yinglan Ye, Hairong Tao, Guotong Dong, Man Hao, Zhijie Zhang, Chunlong Liu

**Affiliations:** 1 Clinical Medical College of Acupuncture Moxibustion and Rehabilitation, Guangzhou University of Chinese Medicine, Guangzhou, China; 2 The First Clinical Medical School, Guangzhou University of Chinese Medicine, Guangzhou, China; 3 Rehabilitation Therapy Center, Luoyang Orthopedic-Traumatological Hospital, Henan Provincial Orthopedic Hospital, Luoyang, China

**Keywords:** flexibility, hamstrings, radial extracorporeal shock wave therapy, rehabilitation, stiffness

## Abstract

**Introduction:**

The hamstrings are essential for sports and daily activities, yet high stiffness and reduced flexibility increase injury risk. Existing relaxation techniques may be ineffective or impractical in clinical settings. This study investigated the immediate effects of radial extracorporeal shock wave therapy (rESWT) on hamstring stiffness and flexibility in individuals with hamstring tightness, providing preliminary evidence for its therapeutic potential for hamstring tightness.

**Methods:**

In total, 131 participants with hamstring tightness were randomized to receive either active rESWT (n = 66) or sham control intervention (n = 65). Flexibility was assessed using the sit-and-reach test (SRT), active knee-extension test (AKE), and active straight leg raising test (ASLR), measured pre-intervention and 5 minutes post-intervention. Muscle stiffness was measured using MyotonPRO at 25% (upper), 50% (middle), and 75% (lower) of the line connecting the ischial tuberosity and the medial/lateral femoral epicondyles, evaluated pre-intervention, immediately post-intervention, and 5 minutes post-intervention.

**Results:**

For flexibility, the rESWT group demonstrated significant, large-effect improvements in SRT (|*r*| = 0.738) and ASLR (|*r*| = 0.848) performance, along with a reduction in knee-extension deficit (AKE, |*d*| = 1.584) (all *P_FDR_* < 0.001). The control group improved significantly only in SRT (*P_FDR_* = 0.008, moderate effect |*r*| = 0.350). Between-group comparisons revealed significantly greater improvements for rESWT in all three measures (all *P_FDR_* < 0.001; moderate-to-large effects: SRT |*δ*| = 0.373, AKE |*d*| = 1.858, ASLR |*δ*| = 0.683). Notably, post-intervention SRT raw scores reflecting final performance showed no significant between-group difference (*P_FDR_* = 0.091). Regarding muscle stiffness, significant group × time interactions with large effects occurred across all hamstring sits (all *P_FDR_* < 0.001, *η²* = 0.200-0.410). Both groups exhibited significantly reduced stiffness immediately post-intervention (all *P_FDR_* < 0.001); however, only the rESWT group maintained significant reductions at 5 minutes post-intervention (all *P_FDR_* < 0.001). The rESWT group exhibited significantly lower stiffness than the controls both immediately and 5 minutes after intervention at the 50%/75% semitendinosus and 75% biceps femoris (all *P_FDR_* < 0.05).

**Conclusion:**

rESWT produces immediate and 5-minute short-term reductions in mid-to-lower hamstring stiffness and improves hamstring flexibility, suggesting potential for the immediate management of hamstring tightness.

## 1 Introduction

The hamstrings are a key muscle group in the posterior aspect of the lower extremity, consisting of the biceps femoris (BF), semitendinosus (ST), and semimembranosus (SM) (Fee et al., 2022). They originate primarily from the ischial tuberosity and insert distally around the head of the fibula and the medial aspect of the tibia. As a biarticular muscle group, the hamstrings cross both the hip and knee joints, and their contraction contributes to hip extension and knee flexion. They play an indispensable role in maintaining postural stability and facilitating daily activities such as walking, climbing stairs, running, jumping, and changing direction, as well as contributing to sports performance (Solomonow et al., 1989). Hamstring injury is one of the most common muscle injuries of the lower extremity, characterized by sudden posterior thigh pain, swelling, and impaired knee flexion and hip extension function (Crupnik et al., 2025). The primary risk factors for hamstring injury include insufficient eccentric strength, excessive accumulation of fatigue, poor muscle flexibility, shortened muscle length, and inadequate warm-up protocols (Liu et al., 2022).

Muscle flexibility is generally defined as the extensibility of the muscle-tendon unit. Greater flexibility improves muscle-tendon compliance, thereby reducing the risk of soft tissue injury caused by excessive range of motion or sudden torsional stress (Witvrouw et al., 2004). In contrast, decreased muscle flexibility leads to increased muscle tone and stiffness, which further decreases the extensibility and functional length of muscles and associated soft tissues, ultimately elevating the risk of muscle strain (Witvrouw et al., 2004). Muscle stiffness, a biomechanical property that reflects resistance to deformation, is inversely associated with muscle flexibility (Raghavan, 2025). Increased muscle stiffness directly compromises flexibility, leading to diminished postural control and impaired athletic performance (Cai et al., 2023). Persistently increased muscle stiffness may predispose individuals to musculoskeletal injuries. Even at rest, increased muscle stiffness may contribute to early fatigue (Sergeeva et al., 2024). Excessive muscle stiffness reduces the compliance of the muscle-tendon unit, impairing the uniform distribution of external mechanical loads within tissues. This results in localized strain concentration, which may induce microstructural damage when strain concentration exceeds the physiological tolerance thresholds of muscle fibers and connective tissues (Fukaya et al., 2022). Such progressive microdamage may accumulate during repetitive loading, thereby increasing the risk of muscle strain. Athletes engaged in high-intensity sports face an elevated risk of hamstring injuries, especially in activities involving sprinting, sudden deceleration, directional changes, and explosive force production, such as soccer and rugby (Freckleton and Pizzari, 2012; Liu et al., 2012). During high-speed, high-intensity exercise, the hamstrings are required to generate forceful contractions in a lengthened position to coordinate hip extension and knee flexion, while repeatedly tolerating high eccentric loads and explosive contractions. Under these conditions, excessive hamstring stiffness may impair the ability of the muscle-tendon unit to elongate, absorb, and dissipate mechanical loads, thereby increasing local strain concentration, accelerating fatigue accumulation, reducing neuromuscular coordination, and ultimately predisposing the hamstrings to strain injury (Heiderscheit et al., 2005; Liu et al., 2012). Additionally, sedentary populations, such as office workers and students who maintain prolonged sitting postures, are also susceptible to hamstring injuries (Dzakpasu et al., 2021). Muscle tone refers to the baseline tension maintained in relaxed skeletal muscle, whereas muscle stiffness reflects the mechanical resistance of muscle tissue to external stretching or deformation (Ganguly et al., 2021). Sustained elevation of muscle tone may increase passive tension within the muscle-tendon unit, thereby contributing to increased muscle stiffness and decreased tissue compliance (Lieber, 1993). During prolonged sitting, the hamstrings remain in a sustained state of passive shortening. This positioning may induce persistent increases in muscle tone and elevate passive tension within the muscle-tendon unit. Chronic maintenance of this state may impair local microcirculation and promote the accumulation of metabolic by-products, thereby compromising tissue recovery and repair (English et al., 2020). These alterations reduce muscle elasticity and compliance, resulting in increased tissue stiffness. Elevated stiffness combined with repetitive mechanical loading during daily activities or athletic performance may facilitate cumulative microdamage, thereby increasing the risk of chronic muscle strain injuries (English et al., 2020; Dzakpasu et al., 2021).

Currently, various preventive strategies for hamstring injuries are available, including static stretching, dynamic stretching, Nordic hamstring exercises, proprioceptive neuromuscular facilitation (PNF) stretching, whole-body vibration (WBV) training, foam rolling, massage, and physical agent therapy (Chaouachi et al., 2015; Houston et al., 2015; Pérez-Bellmunt et al., 2023; Pai et al., 2025). Among these approaches, static stretching is the most widely used; however, it has a relatively slow onset and short-lasting effects (Warneke et al., 2024). Nordic hamstring exercises and PNF stretching can effectively reduce muscle tightness; nevertheless, their effectiveness is highly dependent on participants’ training proficiency and often requires professional supervision (Nunes et al., 2024). Both foam rolling and manual massage may alleviate local muscle stiffness. However, the effectiveness of foam rolling in reducing hamstring stiffness remains controversial, while the outcomes of manual massage are highly dependent on the therapist’s technical expertise and participant cooperation (Warneke et al., 2024). Therefore, identifying an efficient and easily implementable relaxation method with immediate effectiveness is of great significance for rapidly alleviating hamstring tightness, improving flexibility, and optimizing muscle function.

Extracorporeal shock wave therapy (ESWT) is a non-invasive clinical technique that utilizes acoustic pressure waves to deliver mechanical energy to target tissues (Ismail Hassan et al., 2025). These waves transmit energy through rapid compression, coalescence, and high-speed propagation within the transmission medium, ultimately ameliorating pathological alterations in tissues (Sun et al., 2018). In recent years, ESWT has attracted considerable attention in orthopedics, rehabilitation medicine, sports medicine, and other fields (Schroeder et al., 2021; Zhang et al., 2022; De la Corte-Rodríguez et al., 2023; Majidi et al., 2024). Existing studies have demonstrated that ESWT is effective in treating soft tissue injuries, joint stiffness, muscle spasms, and painful conditions (Otero-Luis et al., 2024). Particularly in improving muscle spasticity, an increasing body of clinical evidence indicates that ESWT can effectively alleviate spasticity in patients with stroke, cerebral palsy, multiple sclerosis, and other conditions, thereby enhancing their motor function (Yang et al., 2021). The primary mechanisms of action of extracorporeal shock waves include (Otero-Luis et al., 2024): 1) mechanical stress effect; 2) pressure and cavitation effect; and 3) thermal effect. Based on the energy transmission method, ESWT can be classified into focused extracorporeal shock wave therapy (fESWT) or radial extracorporeal shock wave therapy (rESWT). A systematic review by Dymarek et al. demonstrated that rESWT exhibits superior efficacy in alleviating muscle spasticity compared with fESWT (Dymarek et al., 2020). However, the mechanisms underlying rESWT in reducing muscle stiffness remain incompletely elucidated. One widely accepted hypothesis is “mechanical regulation”, which posits that the mechanical waves generated by rESWT directly act on fibrotic tissue in spastic muscles, reducing intrinsic muscle stiffness, increasing elasticity, releasing soft tissue adhesions, and remodeling collagen fiber alignment, thereby alleviating spasm (d'Agostino et al., 2015; Di et al., 2023). This hypothesis emphasizes that the core mechanism of rESWT in spasm relief lies in altering peripheral muscle mechanical properties rather than spinal neural regulation (d'Agostino et al., 2015). This theoretical mechanism also indicates that rESWT may exert direct biomechanical improvements on non-spastic muscle tension and stiffness. On this basis, we hypothesize that if the therapeutic effect of ESWT on spasticity indeed stems from its direct action on muscle or intramuscular connective tissue, then ESWT may also effectively improve muscle stiffness and flexibility in healthy adults with muscle tightness.

This study recruited young male participants with hamstring tightness and administered rESWT intervention. Using the MyotonPRO device, sit-and-reach test (SRT), active knee-extension test (AKE), and active straight leg raising test (ASLR), the study evaluated the immediate effects of rESWT on hamstring stiffness and flexibility, providing preliminary evidence regarding its potential application as an intervention for improving hamstring tightness.

## 2 Materials and methods

### 2.1 Ethics approval

This study was approved by the Ethics Committee of Shenzhen Dapeng New District Nan’ao People’s Hospital (20250801001) and registered at chictr.org.cn (ChiCTR2600115974) on 4 January 2026. The study was conducted in accordance with the principles of the Declaration of Helsinki. Recruitment commenced following trial registration. Participants were enrolled concurrently for both groups through campus posters and social media advertisements to minimize temporal bias. The formal data collection period spanned from 5 January to 20 March 2026, encompassing the entire enrollment phase. Prior to enrollment, all participants received comprehensive information regarding the safety profiles and potential risks of the MyotonPRO and rESWT devices, their rights, as well as the purpose and detailed procedures of the study. Eligible participants provided written informed consent prior to participation. All participants were instructed to avoid strenuous physical activity for 48 hours before the experiment commenced.

### 2.2 Participants

The sample size was estimated using G*Power 3.1.9.7 (Kang, 2021). The calculation was based on the prespecified primary outcome, defined as the stiffness change from pre-intervention to immediately post-intervention at the dominant side semitendinosus (ST-50%) and biceps femoris (BF-50%) mid-belly sites, and assumed an independent-samples *t*-test to compare these change scores between the rESWT and control groups. Furthermore, although this study measured six hamstring muscle sites, only two predefined sites (ST-50%, BF-50%) were selected for the primary statistical inference, leading to sample size planning that did not account for multiple testing. To mitigate the risk of type I error inflation, all statistical analyses employed false discovery rate (FDR) correction. Given the absence of established reference values for the effect of rESWT on hamstring stiffness, a conservative approach was adopted for sample size estimation. The parameters were set as follows: a medium effect size of Cohen’s d = 0.5, a significance level of α = 0.05, and statistical power = 80% (Heide et al., 2024; Vatovec et al., 2024). The analysis indicated that a minimum of 128 evaluable participants were required, corresponding to 64 participants per group. To account for an anticipated attrition rate of 10%-15%, a total of 146 participants were recruited to ensure an adequate evaluable sample after attrition.

The inclusion criteria were as follows: 1) young males, aged 18–30 years; 2) right-leg dominance, defined as the dominant leg used to generate force during the initial kicking motion; 3) clear consciousness, with the ability to understand the study procedures and cooperate with all testing and intervention protocols; 4) AKE angle >33, which was defined as the knee-extension deficit angle during the AKE test, was considered indicative of hamstring tightness in males (Yıldırım et al., 2018); 5) regular participation in high-intensity sports training or competition (such as football or rugby) for at least 3 consecutive years, with a minimum of 3 training sessions per week (Hosseini et al., 2025); or a mean daily sedentary time of more than 8 hours.

The exclusion criteria were as follows: 1) BMI > 30 kg/m² or BMI < 18.5 kg/m²; 2) previous treatment with rESWT before screening; 3) a history of hamstring strains, lower-limb muscle injuries, or marked pain/discomfort in the lower limbs within the previous 6 months; 4) the presence of neurological disorders, osteoarticular diseases, or other conditions that might affect lower-limb muscle tone or flexibility; 5) any contraindication to rESWT, including local infection, tumors, coagulation disorders, thrombotic diseases, skin ulceration, open wounds, or other skin lesions; 6) current use or injection of medications that might influence the study outcome, such as muscle relaxants, non-steroidal anti-inflammatory drugs, anticoagulants, or botulinum toxin.

### 2.3 Randomization

This study was a randomized controlled trial with participants and outcome assessors blinded. Eligible participants were assigned a unique identification number in the order of enrollment and were randomly allocated using numbers generated by SPSS 27.0 (1 or 2), with 1 indicating the rESWT group and 2 indicating the control group. Group allocation was performed independently by a researcher who was not involved in participant recruitment, intervention administration, or outcome assessment. Participants and outcome assessors were blinded to group allocation throughout the study to minimize potential bias in outcome assessment. Due to the nature of the rESWT intervention, blinding of the therapist administering the intervention was not feasible, as the therapist was required to perform the treatment procedures and therefore had knowledge of the allocation. Thus, only the intervention therapist was unblinded, whereas all participants and outcome assessors remained blinded throughout the study.

### 2.4 Experimental protocol

**Figure 1:**
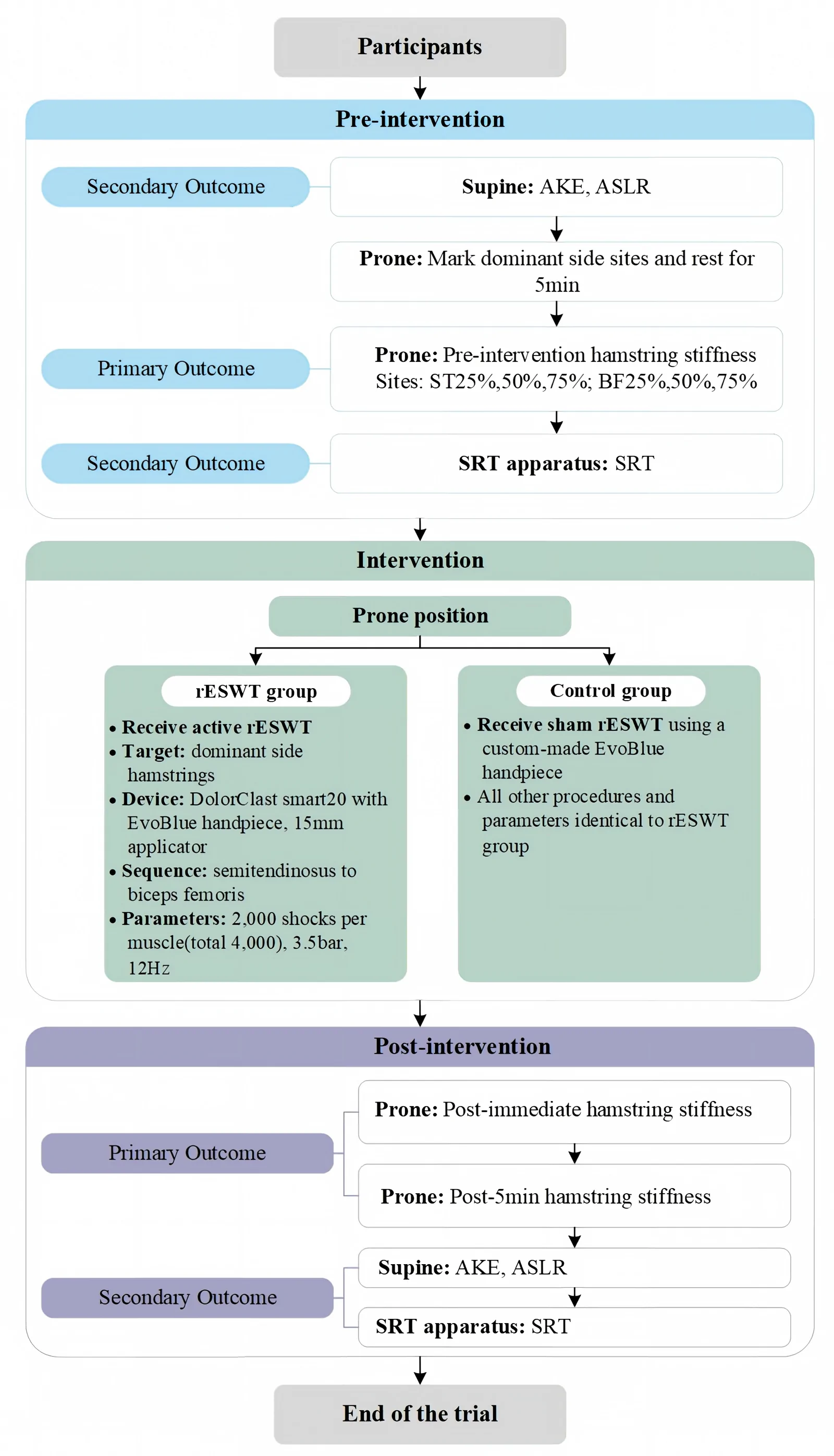
Schematic representation of the experimental protocol. AKE, active knee-extension test; ASLR, active straight leg raising test; SRT, sit-and-reach test; ST, semitendinosus; BF, biceps femoris; rESWT, radial extracorporeal shock wave therapy.

The overall experimental protocol is presented in **Figure 1**. Prior to the intervention, the participants were positioned supine to measure the AKE angle and the ASLR angle. Subsequently, the participants were repositioned to a prone position, and the researcher used an oil-based marker to label measurement sites over the dominant side hamstring regions (specific localization methods are detailed in “Efficacy measures”). Once marking was completed, participants were instructed to maintain the prone position and rest for 5 minutes. After the rest period, pre-intervention hamstring stiffness was measured and recorded at the marked sites using the MyotonPRO (MyotonPRO AS, Estonia), with participants remaining prone throughout data acquisition. Measurements were performed following a medial-to-lateral and upper-to-lower order at the 25%, 50%, and 75% sites along the line from the ischial tuberosity to the medial or lateral femoral epicondyle (hereinafter uniformly referred to as ST-25%, ST-50%, ST-75%, BF-25%, BF-50%, BF-75%). After completing the hamstring stiffness measurements, the participants were guided to the SRT apparatus to collect pre-intervention SRT data.

During the intervention, participants maintained the prone position. A trained physical therapist delivered either active rESWT or sham rESWT to the dominant side hamstring muscles. The instrument used was the pneumatic ballistic extracorporeal shock wave therapy device (DolorClast^®^ smart20, Electro Medical Systems, Nyon, Switzerland), equipped with an EvoBlue handpiece and a 15 mm applicator. Participants in the rESWT group received active rESWT: the therapist slowly moved the applicator from the upper to the lower portion of the muscle belly, sequentially targeting the medial hamstring (ST) and the lateral hamstring (BF), applying 2,000 shocks to each muscle, totaling 4,000 shocks. The treatment parameters were set at a shock wave intensity of 3.5 bar and a frequency of 12 Hz (Schmitz et al., 2015; Crupnik et al., 2025). Participants in the control group received sham rESWT using a custom-made sham EvoBlue handpiece (Crupnik et al., 2025). The sham intervention was performed with identical procedures, including skin contact, anatomical targeting, treatment sequence, treatment duration, and therapist handling. The sham handpiece reproduced the operational sound and superficial mechanical sensations associated with applicator contact and movement but did not deliver therapeutically active radial shock wave impulses (Crupnik et al., 2025). Therefore, the control group was designed to maintain participants’ blinding by mimicking the appearance, sound, and procedural characteristics of active rESWT while eliminating the therapeutic mechanical stimulus.

After the intervention, participants maintained a prone position, and hamstring stiffness data were collected immediately post-intervention and 5 minutes post-intervention. Subsequently, the participants were repositioned to a supine position, and the post-intervention AKE angle and ASLR angle were measured. Finally, participants completed the post-intervention SRT assessment using the SRT apparatus.

### 2.5 Efficacy measures

The primary outcome of this study was hamstring stiffness, which was assessed using the MyotonPRO at three predefined time points: pre-intervention (pre), immediately post-intervention (post-immediate), and 5 minutes post-intervention (post-5min). Secondary outcomes, including hamstring flexibility assessed by the SRT, AKE, and ASLR, were measured at pre-intervention (pre) and 5 min post-intervention (post). Hamstring stiffness values were calculated as the mean of three repeated measurements, whereas peak hamstring flexibility values were determined from the maximum value obtained across three measurements.

To ensure standardization and consistency throughout the trial, two researchers performed distinct, prespecified roles. One researcher was responsible exclusively for administering the active or sham rESWT, whereas the other conducted all outcome assessments, including hamstring stiffness, AKE, ASLR, and SRT. All outcome measurements for all participants were performed by the same assessor, who remained blinded to group allocation throughout the study. Before study initiation, both researchers received standardized training to achieve proficiency in equipment operation and ensure consistent implementation of the experimental protocol.

#### 2.5.1 Hamstring muscle stiffness

**Figure 2:**
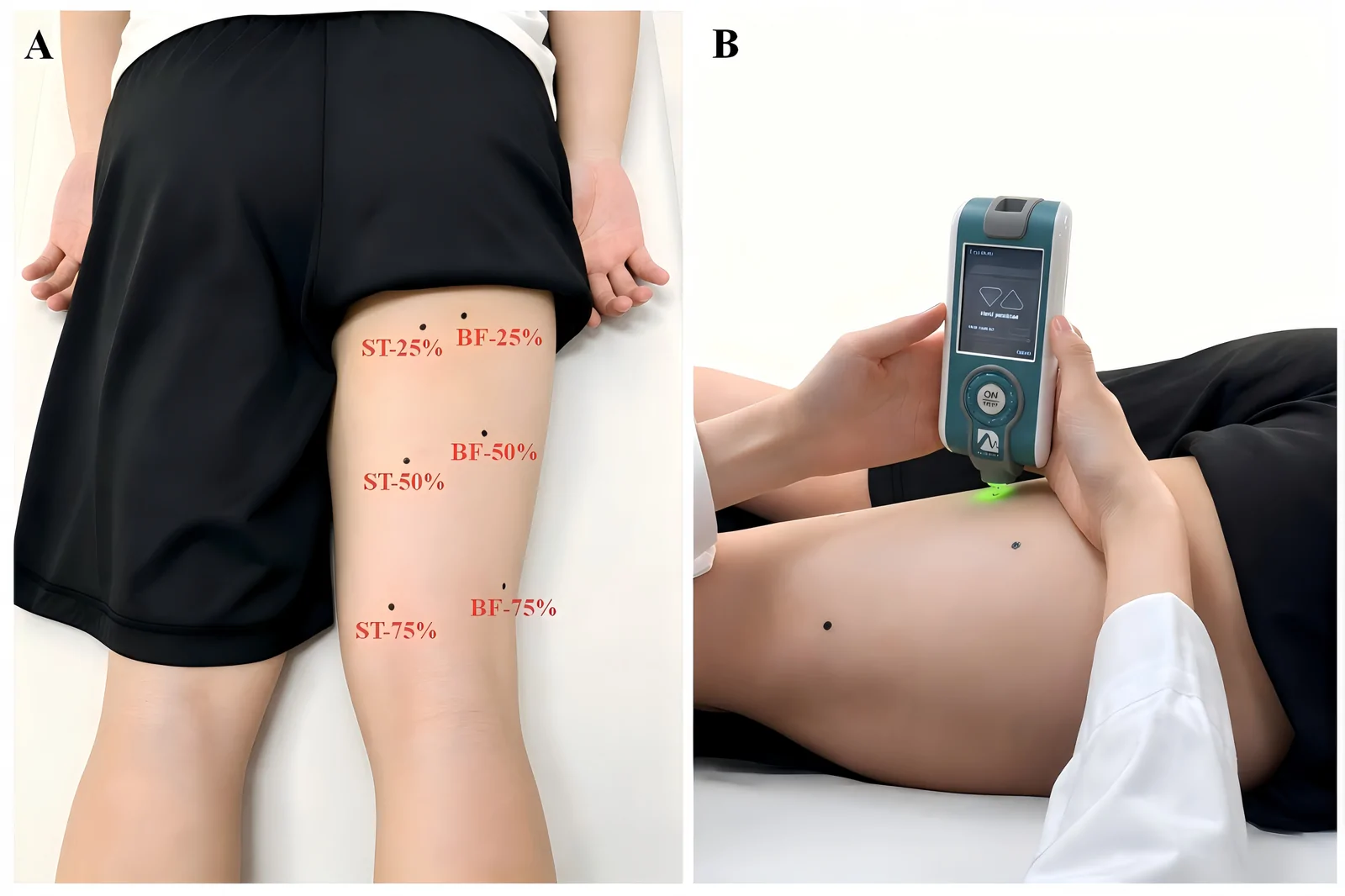
Schematic illustration of hamstring stiffness assessments. **(A)** Anatomical locations of measurement sites on the hamstrings. **(B)** Measurement of hamstring stiffness using the MyotonPRO at the marked sites with the participant in the prone position. ST-25%: 25% point of the line connecting the ischial tuberosity and the medial epicondyle of the femur; ST-50%: midpoint of the line; ST-75%: 75% point of the line. BF-25%: 25% point of the line connecting the ischial tuberosity and the lateral epicondyle of the femur; BF-50%: midpoint of the line; BF-75%: 75% point of the line.

This study utilized the MyotonPRO device to measure hamstring stiffness. Participants assumed a prone position with their hands naturally placed at their sides and ankles relaxed and hanging off the edge of the bed. The assessor positioned the probe vertically on the skin at the marked sites and gently pressed the device until the green indicator light at the probe tip turned on, followed by steadying the instrument. A measurement was completed once the probe rebounded, and the dynamic muscle stiffness (N/m) was displayed on the device screen. Muscle stiffness data were collected at the predefined ST and BF measurement sites (Yagiz et al., 2025). The coefficient of variation (CV) for three consecutive measurements at each site had to be within 3%, and the average value of the three measurements is used as the result for that site. The measurement sites were located as follows (**Figure 2**).

ST-25% (upper part): 25% point of the line connecting the ischial tuberosity and the medial epicondyle of the femur; ST-50% (middle part): midpoint of the line; ST-75% (lower part): 75% point of the line. BF-25% (upper part): 25% point of the line connecting the ischial tuberosity and the lateral epicondyle of the femur; BF-50% (middle part): midpoint of the line; BF-75% (lower part): 75% point of the line.

#### 2.5.2 Hamstring muscle flexibility

**Figure 3:**
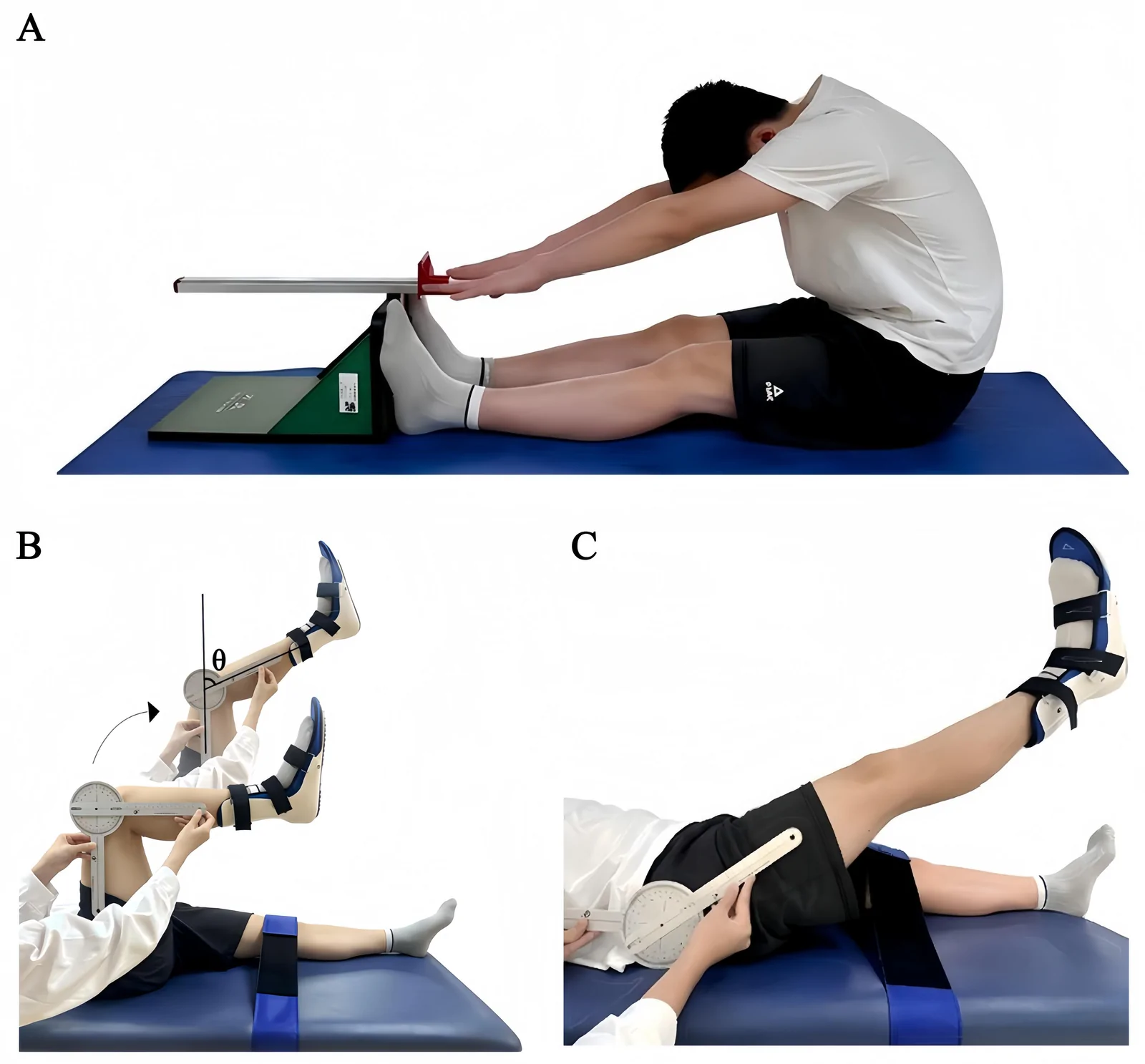
Schematic illustration of hamstring flexibility assessments. **(A)** Sit-and-reach test; **(B)** Active knee-extension test; **(C)** Active straight leg raise test. θ: represents the AKE angle, namely the knee-extension deficit. A greater θ corresponds to poorer hamstring flexibility.

During the SRT (Liu et al., 2022), participants sat barefoot on the testing mat with heels together and toes naturally separated, ensuring that the entire plantar surface of the feet remained vertically flush against the vertical footrest plate of the SRT apparatus. During testing, participants placed their palms downward with both hands parallel and aligned at the middle fingertips, fully extended their arms and torso, and bent their torso forward slowly and steadily. Participants pushed the sliding cursor forward smoothly using the tips of both middle fingers without sudden force, bouncing movements, or unilateral force application. Each test was completed when maximal forward displacement was achieved, and the position was sustained for 2 seconds. Strict supervision was implemented throughout the test: participants kept their knees fully extended, maintained full foot contact with the footrest, and avoided auxiliary trunk force or shoulder shrugging during forward flexion. Higher SRT values indicate better flexibility performance, whereas lower SRT values indicate poorer flexibility performance (**Figure 3A**).

During the AKE (Yıldırım et al., 2018), participants assumed a supine position. The left lower limb was stabilized with a fixation strap, while the right lower limb was held by an experimental assistant at 90° of hip and knee flexion. An ankle brace was used to maintain the participant’s ankle in a neutral position. Participants were instructed to actively extend the right knee with maximal effort until a distinct hamstring stretching sensation was perceived or slight compensatory movement of the left lower limb was observed. At this terminal position, the assessor used a manual goniometer to measure the AKE angle (the goniometer axis was placed at the lateral femoral condyle, the stationary arm aligned parallel to the femoral shaft, and the moving arm aligned parallel to the fibular shaft). The AKE angle was calculated as the difference between full knee-extension (180°) and the knee-flexion angle measured at the maximal active knee-extension position. Therefore, the AKE angle represents the knee-extension deficit. A greater AKE angle corresponds to more severe knee-extension restriction and poorer hamstring flexibility; conversely, a smaller AKE angle indicates a reduction in the knee-extension deficit, less extension restriction and better hamstring flexibility (**Figure 3B**).

During the ASLR (Liu et al., 2022), participants assumed a supine position with both lower limbs naturally extended and the pelvis maintained in a neutral orientation. The left lower limb was stabilized by a fixation strap, and an ankle brace was fitted to maintain ankle neutrality. Participants were instructed to lift the right lower limb actively and slowly while keeping the knee fully extended throughout the motion, until a distinct stretching sensation emerged in the posterior thigh or slight compensatory movement was observed in the left lower limb. At this terminal position, the ASLR angle was recorded (the goniometer axis was placed at the greater trochanter of the femur, with the stationary arm parallel to the longitudinal axis of the trunk and the moving arm parallel to the lateral midline of the femur). Throughout the test, participants were monitored to ensure they maintained proper knee extension. A larger ASLR angle indicated superior hamstring flexibility performance; in contrast, a smaller ASLR angle suggested reduced hamstring flexibility performance (**Figure 3C**).

### 2.6 Statistical analysis

All statistical analyses were performed using SPSS Statistics 27.0 and R 4.6.0. All collected data were first subjected to Shapiro-Wilk normality test and Levene’s test for homogeneity of variances. Data conforming to normality were expressed as mean ± SD, while non-normally distributed data were expressed as median (Q1, Q3). AKE data met the assumptions of normality and homogeneity of variance. Paired-samples *t*-tests were adopted for within-group comparisons, independent-samples t-tests for between-group comparisons, and Cohen’s *d* was calculated to quantify effect sizes. SRT, ASLR and muscle stiffness data violated the normality assumption; accordingly, nonparametric statistical approaches were utilized. For SRT and ASLR data, Wilcoxon signed-rank tests were used for within-group comparisons, and Mann-Whitney *U* tests for between-group comparisons. The effect size *r* was reported for within-group comparisons, and Cliff’s *δ* for between-group comparisons. Nonparametric factorial analysis for mixed designs (nonparametric mixed-design ANOVA) implemented through the nparLD package in R 4.6.0 was applied to muscle stiffness data. When significant main effects or interaction effects were detected, *post-hoc* pairwise comparisons were performed: Wilcoxon rank-sum tests were used for between-group comparisons, and Friedman tests combined with Dunn’s tests for within-group comparisons. Partial *η^2^* was employed to quantify the magnitude of main and interaction effects, while Cliff’s *δ* was reported to estimate effect sizes for all *post-hoc* pairwise comparisons (both within-group and between-group). To control Type I error inflation caused by multiple comparisons, all *P* values were adjusted using the False Discovery Rate (FDR) method. Statistical significance was defined as *P_FDR_* ≤ 0.05. Effect sizes together with their 95% confidence intervals (95%CI), were reported for all comparisons.

## 3 Results

### 3.1 Experimental procedure and baseline data

**Figure 4:**
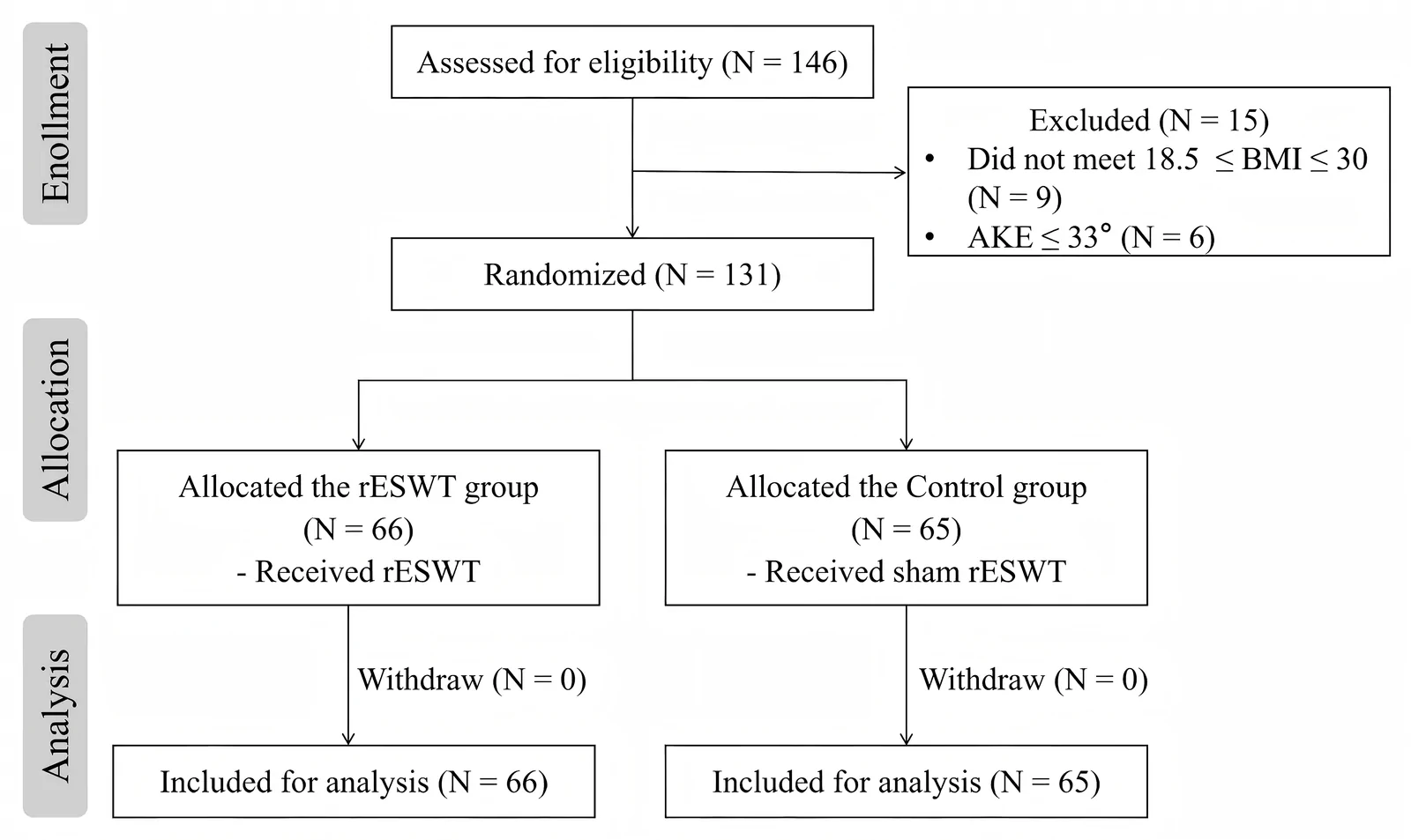
Flowchart of the study. rESWT, radial extracorporeal shock wave therapy; BMI, body mass index. AKE, active knee-extension test.

**Figure 4** illustrates the trial flowchart. A total of 146 participants were recruited for this study, among whom 15 did not meet the inclusion criteria (9 with BMI > 30, 6 with AKE ≤ 33°). Ultimately, 131 participants were randomly assigned to the Control group (n = 65) and the rESWT group (n = 66). No participants dropped out during the trial, and all 131 participants completed the study and were included in the final analysis. No adverse events were observed throughout the experimental protocol. However, adverse events were not systematically assessed using a standardized safety evaluation in this trial. No statistically significant between-group differences were observed in baseline characteristics, including age, height, weight, BMI, activity category (high-activity vs. sedentary), weekly training duration of high-activity participants, and daily sitting time of sedentary participants (all *P* > 0.05), indicating good comparability of baseline data between the groups (**Table 1**).

**Table 1:** Baseline characteristics of participants in the rESWT and control groups.

Characteristics	rESWT group (n = 66)	Control group (n = 65)	Statistical value
Age, years	20.00 (19.00, 20.00)	20.00 (19.00, 21.00)	*Z* = -0.891 *P* = 0.373
Height, cm	176.35 ± 5.54	174.88 ± 6.34	*t* = 1.416 *P* = 0.159
Weight, kg	67.70 ± 6.78	67.18 ± 8.20	t = 0.390 *P* = 0.679
BMI, kg/m^2^	21.74 ± 1.65	21.92 ± 1.94	*t* = -0.549 *P* = 0.584
Activity category, n (%)^†^			*χ²* = 0.071 *P* = 0.789
High-activity participants	32 (48.5%)	30 (46.2%)	
Sedentary participants	34 (51.5%)	35 (53.8%)	
Weekly training duration, h/week^a^	10.50 (9.00, 12.00)	10.50 (9.00, 12.00)	*Z* = -0.695 *P* = 0.487
Daily sitting time, h/day^b^	9.71 ± 0.70	9.63 ± 0.69	*t* = 0.459 *P* = 0.648

Data shown are the mean ± SD, median (Q1, Q3), or n (%) as appropriate. ^†^*P* values were obtained using the Pearson χ² test. ^a^Weekly training duration was analyzed only among high-activity participants (rESWT group, n = 32; control group, n = 30). ^b^Daily sitting time was analyzed only among sedentary participants (rESWT group, n = 34; control group, n = 35). rESWT, radial extracorporeal shock wave therapy; BMI, body mass index.

### 3.2 Changes in hamstring muscle flexibility

The SRT outcomes were presented in **Table 2**. For within-group comparisons, the rESWT group exhibited significant improvement in post-intervention compared to pre-intervention, with a large statistically significant effect size (*P_FDR_* < 0.001, *r* = -0.738, 95%CI [-0.830, -0.608]); the control group also demonstrated a significant increase in post-intervention compared to pre-intervention, with a moderate statistically significant effect size (*P_FDR_* = 0.008, *r* = -0.350, 95%CI [-0.543, 0.122]). For between-group comparisons, change scores (post-pre) yielded a significant difference, indicating that the rESWT group demonstrated a significantly greater improvement in SRT performance than the control group, with a moderate statistically significant effect size (*P_FDR_* = 0.001, *δ* = 0.373, 95%CI [0.213, 0.534]). Nevertheless, no significant between-group difference was detected for post-intervention raw scores, which reflect the final performance level (*P_FDR_* = 0.091). No statistically significant differences were observed between the two groups in pre raw score comparisons (*P_FDR_* = 0.226).

**Table 2:** Changes in SRT pre and post-intervention.

Time/group	rESWT group	Control group	*Z*	*P_FDR_*	Cliff’s *δ*/*r* [95%CI]
Pre	10.80 (5.48, 16.78)	8.70 (3.45, 12.55)	-1.211	0.226	0.123 [-0.048, 0.294]
Post	12.55 (6.38, 17.15)	8.90 (4.25, 13.45)	-1.793	0.091	0.182 [0.012, 0.352]
d (post - pre)	1.25 (0.50, 2.73)	0.50 (-0.20, 1.20)	-3.687	0.001*	0.373 [0.213, 0.534]
Paired rESWT			-5.997	<0.001*	-0.738 [-0.830, -0.608]
Paired Control			-2.820	0.008*	-0.350 [-0.543, 0.122]

Data shown are the median (Q1, Q3). d (post - pre): the post-to-pre intervention change scores. SRT, sit-and-reach test; rESWT, radial extracorporeal shock wave therapy. *P_FDR_ < 0.05. Effect size criteria: small effect |*r*| = 0.1-0.3, |*δ*| = 0.147-0.330; medium effect |*r*| = 0.3-0.5, |*δ*| = 0.330-0.474; large effect |*r*| > 0.5, |*δ*| > 0.474. Effects with 95%CI excluding zero were considered statistically significant.

The AKE outcomes were presented in **Table 3**. Within-group comparisons revealed that the rESWT group exhibited a significant reduction in knee-extension deficit at post-intervention compared with pre-intervention, with a large and statistically significant effect size (*P_FDR_* < 0.001, *d* = -1.584, 95%CI [-1.947, -1.222]). By contrast, no statistically significant difference was observed in the control group between pre-intervention and post-intervention (*P_FDR_* = 0.458). For between-group comparisons, no significant difference was observed between the two groups at pre-intervention (*P_FDR_* = 0.304). After intervention, the rESWT group demonstrated a significantly reduced knee-extension deficit compared with the control group, with a large statistically significant effect size (*P_FDR_* < 0.001, *d* = -1.288, 95%CI [-1.655, -0.912]). The comparison of change scores (post-pre) between the two groups reached statistical significance, indicating that the reduction in the knee-extension deficit was significantly greater in the rESWT group than in the control group, with a large statistically significant effect size (*P_FDR_* < 0.001, *d* = -1.858, 95%CI [-2.268, -1.449]).

**Table 3:** Changes in AKE pre and post-intervention.

Time/group	rESWT group	Control group	*t*	*P_FDR_*	Cohen’s *d* [95%CI]
pre	49.05 ± 8.89	50.85 ± 9.06	-1.147	0.304	-0.201 [-0.544, 0.143]
post	38.70 ± 9.98	51.31 ± 9.58	-7.373	<0.001*	-1.288 [-1.665, -0.912]
d (post - pre)	-10.35 ± 6.53	0.46 ± 4.99	-10.656	<0.001*	-1.858 [-2.268, -1.449]
Paired rESWT			-12.872	<0.001*	-1.584 [-1.947, -1.222]
Paired Control			0.746	0.458	0.093 [-0.151, 0.336]

Data shown are the mean ± SD. d (post - pre): the post-to-pre intervention change scores. AKE, active knee-extension test; rESWT, radial extracorporeal shock wave therapy. *P_FDR_ < 0.05. Effect size criteria: small effect |*d*| = 0.2-0.5; medium effect |*d*| = 0.5-0.8; large effect |*d*| > 0.8. Effects with 95%CI excluding zero were considered statistically significant.

**Figure 5:**
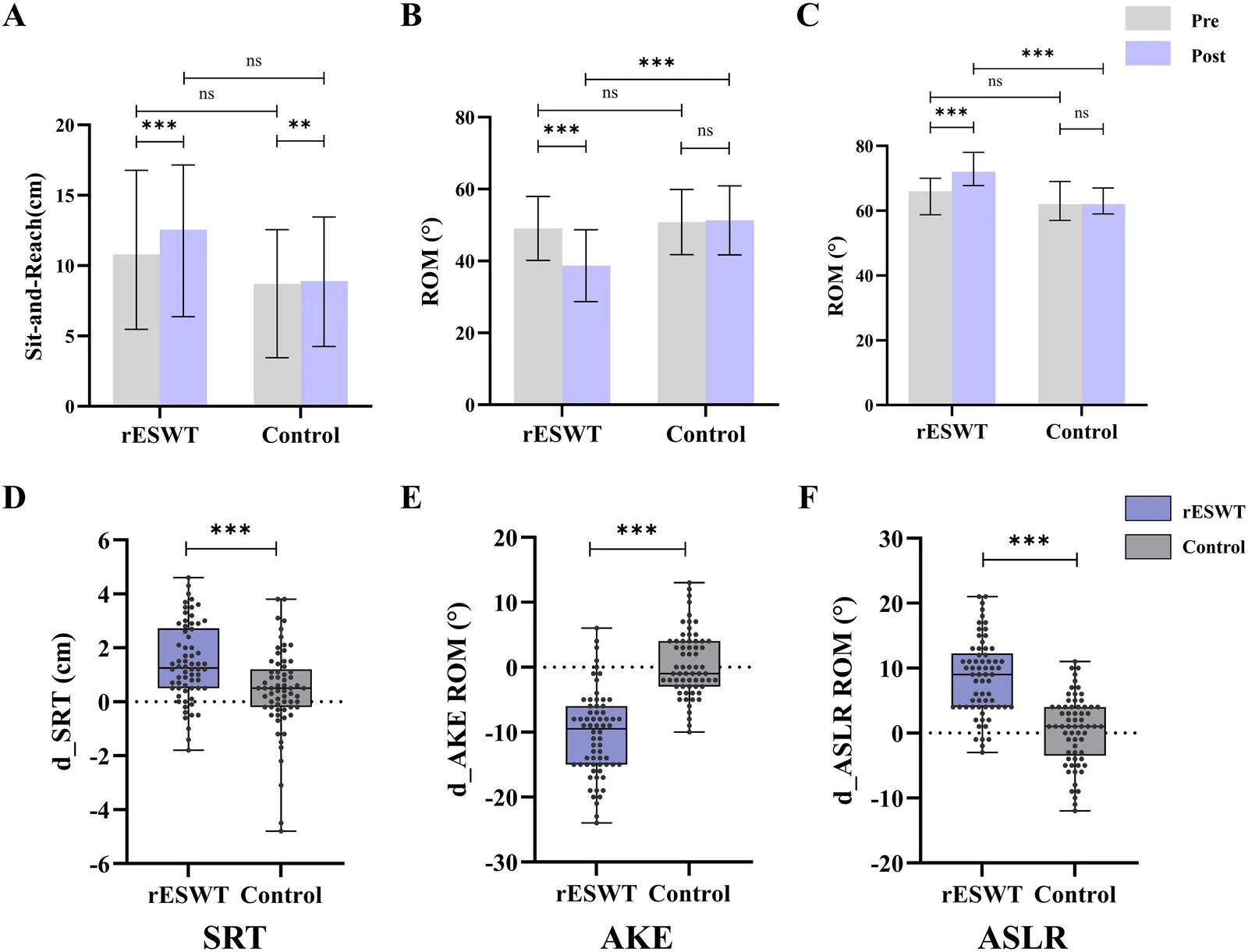
Presents the comparison of the within-group and between-group changes in hamstring flexibility, including SRT, AKE, and ASLR. **(A–C)** show the comparisons between the rESWT and control groups for each measure at pre-intervention and 5 minutes post-intervention, respectively. **(D–F)** show the between-group comparisons of change scores from baseline for SRT, AKE, and ASLR, respectively. rESWT, radial extracorporeal shock wave therapy; SRT, sit-and-reach test; AKE, active knee-extension test; ASLR, active straight leg raise test; ***P < 0.001; **P < 0.01; *P < 0.05; ns, *P* > 0.05.

The ASLR outcomes were presented in **Table 4**. Within-group comparisons revealed that the rESWT group exhibited a significant increase in ASLR angles at post-intervention compared to pre-intervention, with a large and statistically significant effect size (*P_FDR_* < 0.001, *r* = -0.848, 95%CI [-0.904, -0.765]). In contrast, no statistically significant difference was observed in the control group between pre-intervention and post-intervention (*P_FDR_* = 0.349). For between-group comparisons, no significant difference was observed between the two groups at pre-intervention (*P_FDR_* = 0.229); however, post-intervention ASLR angles in the rESWT group were significantly higher than those in the control group, with a large statistically significant effect size (*P_FDR_* < 0.001, *δ* = 0.597, 95%CI [0.458, 0.735]). The between-group comparison of change scores (post-pre) reached statistical significance, indicating that the rESWT group demonstrated a significantly greater improvement in ASLR performance than the control group, with a large, statistically significant effect size (*P_FDR_ <* 0.001, *δ* = 0.683 [0.556, 0.809]). The within-group and between-group changes in hamstring flexibility, as assessed by SRT, AKE, and ASLR, are presented in **Figure 5**.

**Table 4:** Changes in ASLR pre and post-intervention.

Time/group	rESWT group	Control group	*Z*	*P_FDR_*	Cliff’s *δ*/*r* [95%CI]
Pre	66.00 (58.75, 70.00)	62.00 (57.00, 69.00)	-1.332	0.229	0.135 [-0.036, 0.229]
Post	72.00 (67.75, 78.00)	62.00 (59.00, 67.00)	-5.894	<0.001*	0.597 [0.458, 0.735]
d (post - pre)	9.00 (4.00, 12.25)	1.00 (-3.50, 4.00)	-6.751	<0.001*	0.683 [0.556, 0.809]
Paired rESWT			-6.892	<0.001*	-0.848 [-0.904, -0.765]
Paired Control			-0.936	0.349	-0.116 [-0.345, 0.126]

Data shown are the median (Q1, Q3). d (post - pre): the post-to-pre intervention change scores. ASLR, active straight leg raise test; rESWT, radial extracorporeal shock wave therapy. *P_FDR_ < 0.05. Effect size criteria: small effect |*r*| = 0.1-0.3, |*δ*| = 0.147-0.330; medium effect |*r*| = 0.3-0.5, |*δ*| = 0.330-0.474; large effect |*r*| > 0.5, |*δ*| > 0.474. Effects with 95%CI excluding zero were considered statistically significant.

### 3.3 Changes in hamstring muscle stiffness

Main effects of group, time, and their interaction on hamstring muscle stiffness from the repeated-measures model, as well as detailed *post-hoc* results, are summarized in **Supplementary Materials S1** and **S2**. Significant main time effects with large effect sizes were identified for muscle stiffness at all ST and BF measurement sites (all *P_FDR_* < 0.001, *η²* = 0.282-0.482). A significant moderate main group effect was only found for stiffness at the ST 50% site (*P_FDR_* = 0.015, *η²* = 0.048). Notably, significant Group × Time interaction effects with large effect sizes were detected for muscle stiffness across all ST and BF regions (all *P_FDR_* < 0.001, *η²* = 0.200-0.410), demonstrating divergent temporal changes in muscle stiffness between groups. *Post-hoc* within- and between-group comparisons were subsequently performed. *Post-hoc* analyses for the ST and BF muscles are presented in **Tables 5** and **6**.

**Table 5:** Changes in semitendinosus muscle stiffness at three sites: pre, post-immediate, and post-5min intervention.

Site	Time	rESWT group	Control group	*Z*	*P_FDR_*	Cliff’s *δ* [95%CI]
ST-25%	Pre	239.17 (198.42, 277.83)	220.00 (186.17, 263.50)	-1.093	0.367	0.111 [-0.061, 0.282]
	Post-immediate	202.00 (167.00, 243.83)^a^	213.33 (179.33, 255.50)^a^	-1.620	0.189	-0.164 [-0.335, 0.006]
	Post-5min	216.83 (172.92, 257.33)^a^	219.00 (184.83, 258.33)	-1.068	0.367	-0.108 [-0.280, 0.063]
	*χ^2^/P_FDR_*	95.176/<0.001*	29.566/<0.001*	/	/	/
ST-25%	Pre	326.33 (308.75, 353.75)	327.67 (309.83, 344.50)	-0.364	0.716	0.037 [-0.135, 0.210]
	Post-immediate	300.50 (281.17, 311.50)^a^	318.00 (307.33, 343.00)^a^	-4.185	<0.001*	-0.424 [-0.580, -0.268]
	Post-5min	304.67 (287.42, 321.42)^a^	326.67 (307.83, 348.33)	-3.998	<0.001*	-0.405 [-0.563, -0.247]
	*χ^2^/P_FDR_*	97.331/<0.001*	44.116/<0.001*	/	/	/
ST-75%	Pre	384.17 (347.25, 437.67)	367.67 (336.50, 409.00)	-0.859	0.439	0.087 [-0.085, 0.259]
	Post-immediate	339.67 (304.58, 396.83)^a^	360.33 (332.00, 400.33)^a^	-2.465	0.031*	-0.250 [-0.417, -0.082]
	Post-5min	347.00 (304.00, 397.83)^a^	365.33 (336.33, 413.17)	-2.550	0.031*	-0.259 [-0.425, -0.091]
	*χ^2^/P_FDR_*	83.336/<0.001*	20.901/<0.001*	/	/	/

Data shown are the median (Q1, Q3). In the within-group comparison, ^a^indicates comparison with pre, *P_FDR_* < 0.05. rESWT, radial extracorporeal shock wave therapy; ST, semitendinosus; 25%, 50%, 75%: measured at 25%, 50%, 75% from the ischial tuberosity to the medial femoral epicondyles. *P_FDR_ < 0.05. Effect size criteria: small effect |*δ*| = 0.147-0.330; medium effect |*δ*| = 0.330-0.474; large effect |*δ*| > 0.474. Effects with 95%CI excluding zero were considered statistically significant.

**Table 6:** Changes in biceps femoris muscle stiffness at three sites: pre, post-immediate, and post-5min intervention.

Site	Time	rESWT group	Control group	*Z*	*P_FDR_*	Cliff’s *δ* [95%CI]
BF-25%	Pre	210.17 (171.17, 264.17)	194.00 (176.17, 235.83)	-0.725	0.527	0.074 [-0.098, 0.246]
	Post-immediate	178.50 (151.00, 238.67)^a^	198.00 (175.83, 235.50)^a^	-1.809	0.127	-0.183 [-0.353, -0.013]
	Post-5min	190.67 (153.08, 242.33)^a^	201.00 (178.00, 237.67)	-1.607	0.162	-0.163 [-0.333, 0.007]
	*χ^2^/P_FDR_*	89.576/<0.001*	19.129/<0.001*	/	/	/
BF-25%	Pre	334.33 (314.42, 351.42)	324.33 (304.00, 346.67)	-1.040	0.383	0.106 [-0.066, 0.277]
	Post-immediate	307.50 (277.08, 327.25)^a^	319.33 (301.33, 343.00)^a^	-2.334	0.059	-0.237 [-0.404, -0.069]
	Post-5min	313.00 (285.92, 334.33)^a^	326.00 (303.83, 345.50)	-3.998	0.062	-0.223 [-0.391, -0.055]
	*χ^2^/P_FDR_*	90.411/<0.001*	21.953/<0.001*	/	/	/
BF-75%	Pre	418.17 (389.17, 485.25)	431.67 (398.50, 460.83)	-0.859	0.991	0.001 [-0.171, 0.174]
	Post-immediate	389.00 (363.75, 447.33)^a^	421.67 (393.83, 459.00)^a^	-2.465	0.029*	-0.277 [-0.443, -0.111]
	Post-5min	394.67 (364.17, 446.33)^a^	426.00 (397.83, 461.50)	-2.550	0.029*	-0.290 [-0.455, -0.124]
	*χ^2^/P_FDR_*	69.252/<0.001*	14.395/<0.001*	/	/	/

Data shown are the median (Q1, Q3). In the within-group comparison, ^a^indicates comparison with pre, *P_FDR_* < 0.05. rESWT, radial extracorporeal shock wave therapy; BF, biceps femoris; 25%, 50%, 75%: measured at 25%, 50%, 75% from the ischial tuberosity to the lateral femoral epicondyles. *P_FDR_ < 0.05. Effect size criteria: small effect |*δ*| = 0.147-0.330; medium effect |*δ*| = 0.330-0.474; large effect |*δ*| > 0.474. Effects with 95%CI excluding zero were considered statistically significant.

Within-group comparisons revealed that in the rESWT group, muscle stiffness at ST-25%, ST-50%, ST-75%, BF-25%, BF-50% and BF-75% was significantly reduced at post-immediate (respectively ST: all *P_FDR_* < 0.001, |*δ*| = 0.864-0.939; BF: all *P_FDR_* < 0.001, |*δ*| = 0.848-0.939) and post-5min (respectively ST: all *P_FDR_* < 0.001, |*δ*| = 0.848-0.985; BF: all *P_FDR_* < 0.001, |*δ*| = 0.788-0.909) relative to pre. All comparisons yielded large effect sizes, and the corresponding 95%CIs indicated statistically significant effects. In the control group, muscle stiffness at ST-25%, ST-50%, ST-75%, BF-25%, BF-50%, and BF-75% exhibited a significant decrease only at post-immediate compared with pre (respectively ST: *P_FDR_* < 0.001, |*δ*| = 0.492-0.615; BF: all *P_FDR_* < 0.002, |*δ*| = 0.323-0.415). Effect sizes ranged from moderate to large for the three ST sites and from small to large for the three BF sites. By contrast, no statistically significant differences in muscle stiffness were detected between pre and post-5min for all measured sites (all *P_FDR_* > 0.05).

**Figure 6:**
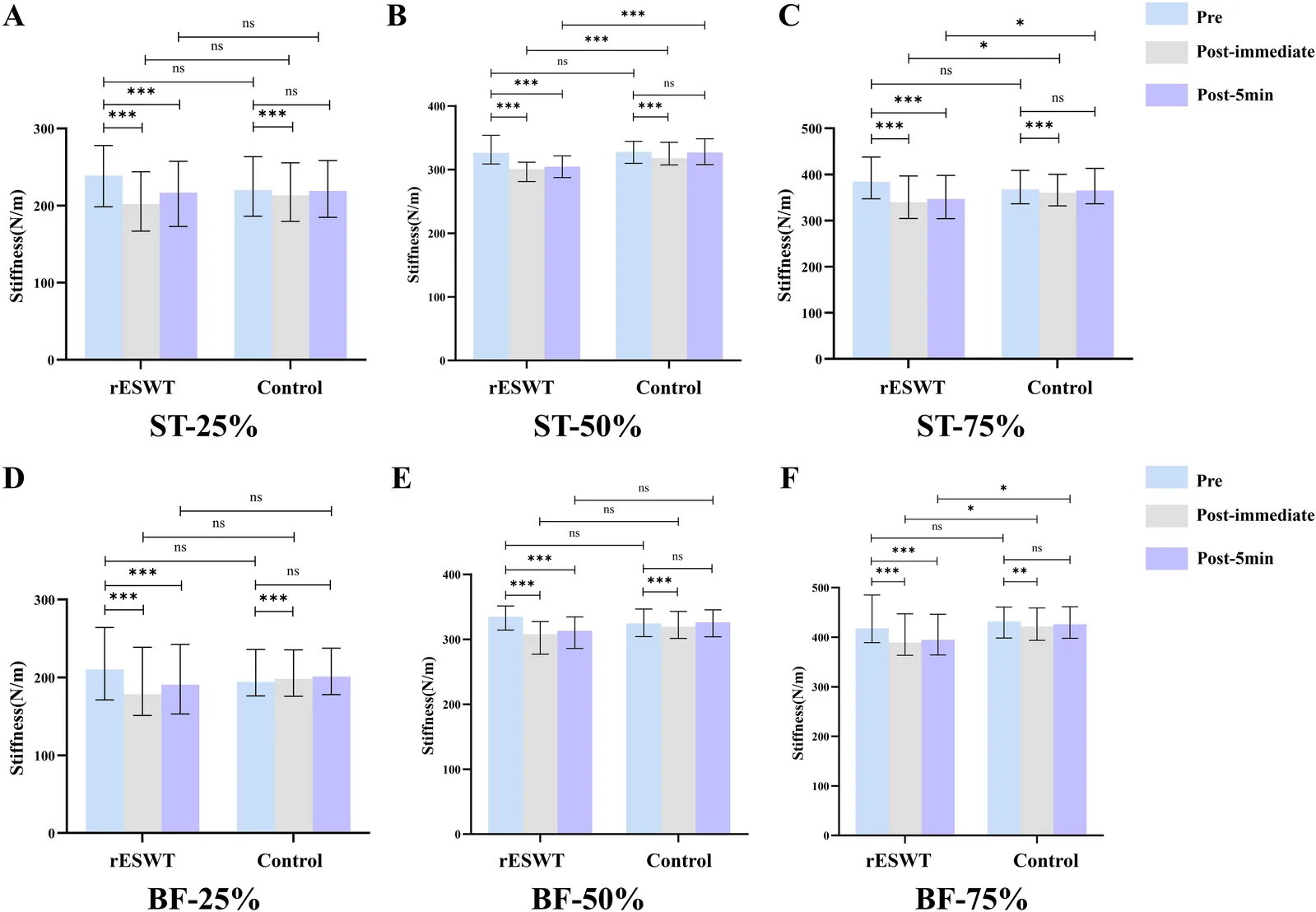
Presents the comparison of the within-group and between-group changes in hamstring stiffness at pre-intervention, immediately post-intervention and 5 min post-intervention. **(A–C)** Statistical comparative analysis results of ST-25%, ST-50% and ST-75%, respectively. **(D–F)** Statistical comparative analysis results of BF-25%, BF-50% and BF-75%, respectively. ST, semitendinosus; BF, biceps femoris. 25%, 50%, 75%: measured at 25%, 50%, and 75% from the ischial tuberosity to the medial/lateral femoral epicondyles; ***P < 0.001; **P < 0.01; *P < 0.05; ns, *P* > 0.05.

Between-group comparisons showed no significant differences in muscle stiffness at ST-25%, BF-25% and BF-50% between groups at pre, post-immediate and post-5min (all *P_FDR_* > 0.05). At ST-50%, muscle stiffness was markedly lower in the rESWT group versus the control group at post-immediate and post-5min, with moderate statistically significant effect sizes (both *P_FDR_* < 0.001, |*δ*| = 0.405-0.424). For ST-75% and BF-75%, significantly lower muscle stiffness was observed in the rESWT group at post-immediate (respectively ST-75%: *P_FDR_* = 0.031, *δ* = -0.250, 95%CI [-0.417, -0.082]; BF-75%: *P_FDR_* = 0.029, *δ* = -0.277, 95%CI [-0.443, -0.111]) and post-5min (respectively ST-75%: *P_FDR_* = 0.031, *δ* = -0.259, 95%CI [-0.425, -0.091]; BF-75%: *P_FDR_* = 0.029, *δ* = -0.290, 95%CI [-0.455, -0.124]). Effect sizes for these comparisons were small, and all corresponding 95%CIs confirmed statistical significance. The within-group and between-group changes in hamstring stiffness, measured at pre-intervention, immediately post-intervention, and 5 min post-intervention, are presented in **Figure 6**.

## 4 Discussion

This study aimed to investigate the immediate effects of a single session of rESWT in young males with hamstring tightness, focusing on its influences on hamstring stiffness and flexibility. A randomized, sham-controlled design was employed, in which participants received either active rESWT or sham rESWT. Assessments were performed at pre-intervention, immediately post-intervention, and 5 minutes post-intervention. Hamstring stiffness was evaluated at the 25%, 50%, and 75% sites of the ST and BF, whereas flexibility outcomes were assessed using the SRT, AKE, and ASLR. The results showed that a single session of rESWT significantly reduced stiffness at the mid-to-lower hamstring regions, with effects persisting up to 5 minutes post-intervention. Furthermore, rESWT produced substantial improvements in hamstring flexibility, as reflected by SRT, AKE, and ASLR. Although the control group also exhibited reduced muscle stiffness immediately post-intervention, these effects returned to baseline levels after 5 minutes. These findings suggest that rESWT may modulate hamstring mechanical properties and flexibility-related outcomes. However, as observations are limited to the period from immediate post-intervention to 5 minutes post-intervention following one single treatment session, long-term effects of repeated rESWT remain to be established, and the present results only offer preliminary evidence supporting the use of this therapy for relieving muscle tightness.

### 4.1 The improvement effect of rESWT on hamstring muscle flexibility

Insufficient flexibility is often considered to be directly related to athletic performance and injury risk (Opar et al., 2012). This study utilized three indicators (SRT, AKE, and ASLR) to evaluate the intervention effects of rESWT on hamstring flexibility, with the results demonstrating significant differences.

The SRT reflects the overall flexibility of the spine-pelvis-posterior lower limb muscle groups (Mayorga-Vega et al., 2014). SRT was used to evaluate overall flexibility, and the results showed that both the rESWT and control groups exhibited significant improvements compared with baseline levels after the intervention. Between-group comparisons of the raw SRT scores before and after intervention revealed no statistically significant differences. Critically, while post-intervention raw SRT scores did not differ significantly between groups, the rESWT group demonstrated significantly greater improvement in change scores (post-pre) than the control group, with a moderate effect size. This divergence arises because raw score comparisons reflect final performance levels, whereas change scores focus on the magnitude of improvement from baseline. Therefore, although rESWT may enhance the trajectory of SRT improvement, both groups achieved similar final performance levels. Consequently, these two analytical strategies can produce divergent statistical results. Even though the larger improvement observed in the rESWT group suggests that rESWT may confer beneficial effects on SRT performance. Nevertheless, the interpretation of SRT findings should be approached with caution. Firstly, rESWT was applied only to the dominant side hamstrings in this study, whereas SRT performance depends on the coordinated contribution of bilateral hamstrings. Secondly, SRT is a composite flexibility assessment that is influenced not only by hamstring extensibility but also by lumbar spine mobility, pelvic movement capacity, and coordinated motion of the posterior kinetic chain (Mayorga-Vega et al., 2014). Therefore, although the rESWT group demonstrated a greater improvement in SRT performance, the extent to which this improvement can be attributed specifically to enhanced flexibility of the treated hamstrings remains unclear.

Multiple previous studies have raised controversies regarding the validity of the SRT as a tool for assessing hamstring flexibility. A meta-analysis conducted by Mayorga-Vega et al. (2014) systematically evaluated the validity of various SRT in assessing the flexibility of the hamstrings and lumbar spine. The results indicated that the SRT demonstrated moderate validity (rp = 0.46-0.67) for hamstring flexibility and low validity (rp = 0.16-0.35) for lumbar spine flexibility. Among the tests, the classic SRT (compared to the modified finger-to-box test) was identified as the optimal method for assessing hamstring flexibility, with higher validity observed in females and populations with higher flexibility levels. However, Muyor et al. (2014) suggested that the SRT is significantly influenced by anterior pelvic tilt and lumbar flexion, making it an effective measurement tool for evaluating spinal flexibility and the range of pelvic tilt motion. And its validity for assessing a single localized muscle group (the hamstrings) is very low. Compared with gold standard tests such as AKE or ASLR, the SRT shows only low to moderate correlation and cannot accurately reflect the extensibility of the hamstrings (Cameron and Bohannon, 1993; Neto et al., 2015). Moreover, gender differences were found to influence the results of the SRT. Mier (2011) used video analysis technology to evaluate the hamstring flexibility and the validity of the SRT in 60 adult men and women. The results showed that, using the PSLR test as a criterion, the SRT demonstrated high validity for hamstring flexibility in women (R = 0.81), but only moderate validity in men (R = 0.66).

In conclusion, all participants recruited in this study were young males with hamstring tightness. Prior studies have suggested that the validity of the SRT is modulated by sex and flexibility level. Therefore, the interpretation of SRT results in this study requires cautious analysis in combination with participant characteristics or more targeted hamstring flexibility assessments (AKE or ASLR).

The AKE and ASLR are the two most commonly used methods for clinically evaluating hamstring flexibility (Allam et al., 2023). The AKE involves fixing the hip joint at 90° of flexion and requiring participants to actively extend the knee. This test focuses solely on knee-extension and directly reflects the extensibility of the hamstring muscles, with minimal interference from other factors (Reurink et al., 2013). In contrast, the ASLR requires participants to actively flex the hip while keeping the knee fully extended. The results of this test are not only influenced by hamstring flexibility but are also affected by multiple factors, such as hip-flexor muscle strength, lumbar compensation, and pelvic stability (Hu et al., 2012). In this study, the results of the AKE and ASLR demonstrated a high degree of consistency, specifically manifested as follows: within-group comparisons revealed a significant reduction in the knee-extension deficit (AKE) and a significant improvement in ASLR angle relative to baseline in the rESWT group, while no significant changes were observed in the control group before and after the intervention. Between-group comparisons indicated statistically significant differences in both raw scores and change scores between the two groups, with large effect sizes. Collectively, these findings confirm that rESWT can effectively improve the immediate hamstring flexibility of participants. This conclusion is consistent with the findings of Kim et al. (2017) That study enrolled 29 healthy adults with hamstring tightness to investigate the immediate effects of rESWT on hamstring flexibility, assessed through the finger-to-floor test and passive straight leg raise test (PSLR). The results demonstrated that rESWT significantly alleviated hamstring tightness in healthy individuals immediately after intervention (Kim et al., 2017). This rapid therapeutic response may suggest that improved hamstring muscle flexibility stems primarily from direct biomechanical effects of rESWT. Specifically, mechanical vibrations may transiently alter the mechanical properties of muscle tissue and local circulation, potentially contributing to an immediate reduction in perceived muscle tension rather than relying solely on delayed mechanotransduction effects, which involve time-dependent biological responses that may not become apparent immediately (Di et al., 2023). Moreover, the results of the AKE and ASLR exhibited good consistency, which supports the conclusions of previous related studies. Cameron and Bohannon (1993) selected 23 healthy adults as research participants, used a 35-mm camera to record joint range of motion, and analyzed the reliability and correlation of AKE and ASLR in assessing hamstring flexibility. They found that both AKE (ICC = 0.861) and ASLR (ICC = 0.953) exhibited good test-retest reliability, and the results of the two tests showed a significant negative correlation (r = -0.718, *P* < 0.001), indicating that both can serve as effective indicators for assessing hamstring flexibility. Neto et al. (2015) conducted a reliability analysis targeting 102 recreational athletes with poor hamstring flexibility, systematically investigating the test-retest reliability of the AKE and ASLR, as well as the correlation between them. Their findings indicated that both assessment methods exhibited excellent measurement reliability (AKE ICC = 0.87-0.94, ASLR ICC = 0.93-0.97) and there was a moderate to high significant correlation (dominant limb r = 0.71, nondominant limb r = 0.67) between the results of the two tests, confirming that both the AKE and ASLR can serve as stable and reliable evaluation tools for hamstring flexibility (Neto et al., 2015). The findings of this study indicate that rESWT significantly reduced the knee-extension deficit (AKE) and increased ASLR angles, suggesting that rESWT may effectively improve immediate hamstring flexibility.

### 4.2 The improvement effect of rESWT on hamstring muscle stiffness

Muscle mechanical properties are a key factor determining athletic performance (de Paula Simola et al., 2016). Excessively high muscle stiffness not only reduces athletic performance but also significantly increases the risk of sports injuries such as muscle strains and tears (Watsford et al., 2010). As a non-invasive musculoskeletal rehabilitation therapy, rESWT is widely used in sports injury rehabilitation. Cacchio et al. (2010) investigated ESWT in 40 professional athletes with chronic proximal hamstring tendinopathy and found that ESWT improved pain and functional outcomes in these athletes. At the 3-month follow-up, 85% of the patients achieved at least 50% pain relief, and 80% of the patients returned to their pre-injury level of sports activity, indicating that ESWT potentially contributes to the recovery of hamstring-related motor function. The study by Crupnik et al. (2025) found that rESWT combined with a structured rehabilitation program was associated with a shorter return-to-sport time and potentially promoted the recovery of lower limb muscle strength in athletes with acute type 3b hamstring injuries. Its mechanism of action may be closely linked to direct improvement of muscle mechanical properties. Morgan et al. (2021) found that rESWT combined with multimodal therapy might shorten the time away from competition among elite football players with acute type 1a, 2b, and 3a muscle injuries, lower reinjury risk, and limit the progression of structural injuries (e.g., muscle tightness, soreness).

This study is the first to investigate the immediate effects of rESWT on muscle stiffness in different segments of the hamstring muscles in young males with hamstring tightness. Nonparametric mixed-design ANOVA revealed a significant and substantial group-by-time interaction effect for muscle stiffness results across all measured hamstring sites, indicating divergent trajectories of muscle stiffness change over time between the two subject groups. Within-group analyses revealed that hamstring muscle stiffness decreased at all measurement sites immediately post-intervention in both the rESWT and control groups. However, hamstring muscle stiffness in the control group returned to baseline at 5 minutes post-intervention. These findings suggest that the reduction in hamstring stiffness observed immediately after treatment may be affected by transient extraneous factors independent of rESWT (e.g., temporal effects, postural adaptation, placebo responses). In contrast, the improvement in hamstring stiffness observed in the rESWT group persisted until 5 minutes post-intervention. Critically, between-group comparisons revealed that the magnitude of hamstring stiffness reduction was significantly greater compared with the control group, particularly at measurement sites corresponding to 50% ST, 75% ST, and 75% BF. These localized short-term sustained effects, observed both immediately post-intervention and 5 minutes post-intervention, provide preliminary evidence for the mechanical action exerted by rESWT on muscle tissue.

The findings of this study can provide supplementary support for the conclusions of previous related research. Sohn et al. (2011) reported a reduction in Modified Ashworth Scale scores in patients with post-stroke gastrocnemius spasticity following rESWT, without corresponding changes in electrophysiological measures (F-wave, H-reflex, and tibial conduction), indicating that its anti-spastic mechanism likely involves peripheral mechanical adaptations rather than spinal excitability modulation. Leng et al. (2021) investigated patients with post-stroke wrist spasticity and found that rESWT could immediately reduce the viscous component of muscle tissue, muscle tone, and stiffness. These results suggest that rESWT may have a more pronounced effect on improving the mechanical properties of peripheral muscles. Its mechanical action possibly reduces muscle fibrosis and alters the rheological properties (viscosity and stiffness) of spastic muscles. Lee et al. (2019) investigated the effects of a single session of ESWT on gastrocnemius muscle spasm in patients with chronic stroke through ultrasound evaluation. These observations suggest that ESWT may improve connective tissue fibrosis and abnormal muscle architecture within chronically hypertonic muscles primarily through direct mechanical effects, rather than predominantly depending on neuromodulatory mechanisms.

It is noteworthy that no significant between-group differences in hamstring stiffness were observed in the upper part of the hamstrings (ST-25%, BF-25%) or the middle part of the biceps femoris (BF-50%) in this study. This phenomenon may be related to variations in baseline muscle stiffness levels across different anatomical sites, as well as differences in the extent of changes induced by mechanical stimulation across these muscle regions. Firstly, the results of this study indicate that, for both ST and BF, muscle stiffness generally exhibits an increasing gradient from the upper to lower, with the upper part < middle part < lower part. When the baseline stiffness of a specific region is relatively low, even if rESWT exerts a mechanical effect in the same direction, the absolute magnitude of the reduction remains relatively limited, which may result in a lack of statistically significant differences. This result is consistent with the research findings of Miyamoto et al. (2020), wherein shear wave elastography measurements during hamstring lengthening (induced by knee-extension or hip-flexion) revealed a significantly higher passive muscle shear modulus in the lower versus upper regions across all examined muscles (BF, ST, and SM). That is, the lower region had a higher initial stiffness level. Therefore, although the relative decrease in shear modulus between the upper and lower regions of the hamstring muscles might be similar, due to the higher baseline stiffness value in the lower region, the absolute decrease is also usually greater than that in the upper region, which may consequently lead to an insignificant difference in the between-group comparison of the upper hamstring regions. Secondly, there were no significant between-group differences at the BF-50% site, possibly suggesting that different hamstring components show varying reactivity to mechanical stimulation. Kellis (2018) indicated that the upper and lower portions of the hamstrings belong to different mechanical functional units. This implies that a single intervention may struggle to elicit consistent mechanical effects along the entire length of the muscle. Zhang et al. assessed the mechanical changes of hamstrings during isometric trunk extension at different intensities using MyotonPRO and observed that the stiffness changes in ST and BF were not fully aligned. Across various load levels, ST tended to exhibit a larger stiffness response compared with BF, which may suggest divergent mechanical responsiveness. The authors speculated that this phenomenon could be associated with differences in muscle architecture, fiber-type distribution, and tissue compliance (Zhang et al., 2023). Therefore, in this study, even when rESWT with identical parameters was directly applied to both ST and BF, different parts of the hamstrings may still show differential mechanical responses, which could account for the lack of significant difference at BF-50%. Additionally, although MyotonPRO has been demonstrated to have good measurement reliability in previous studies (Chen et al., 2019; Lettner et al., 2024), the relatively thick subcutaneous adipose tissue in the upper hamstrings may attenuate the energy of the measurement impulse and affect the sensitivity of stiffness detection (Ley et al., 2025). Therefore, although rESWT may have had a real effect on the stiffness of the upper muscle, MyotonPRO might not have been able to detect the difference from the control group due to interference from the superficial fat.

## 5 Limitations

This study has several limitations. Firstly, the observation period was relatively short, with only three time points set: pre-intervention, immediately post-intervention, and 5 minutes post-intervention. Therefore, the results primarily reflect the immediate effects of rESWT on the mechanical properties of the hamstrings, and it is not yet possible to infer its sustained effects or long-term intervention value based on these findings. Secondly, this study used MyotonPRO to measure different sites (25%, 50%, and 75%) of the hamstrings. This method primarily reflects the dynamic mechanical properties of local superficial soft tissues. Although it offers good convenience and repeatability, the results may still be influenced by factors such as measurement point positioning, local soft tissue thickness, the composition of the myofascial tissue, and the complex upper anatomical structure. Particularly in the 25% region of the hamstrings, these influences may further increase measurement variability, thereby diminishing the ability to detect between-group differences. Thirdly, the experimental protocol involved multiple changes in participant positioning, including transitions between supine and prone positions for different outcome assessments. Although these procedures were standardized and implemented consistently across all participants, postural transitions may alter muscle length and passive tension, thereby introducing subtle effects on measurements of hamstring stiffness and flexibility. Additionally, we performed stratified *post-hoc* comparisons of muscle stiffness data to characterize both between-group differences at individual time points and within-group changes over time, aligning with our primary aim of exploring the short-term time-dependent effects of the rESWT intervention. However, these analyses constituted independent tests rather than model-derived contrasts and failed to utilize the full dataset. Despite multiple comparison corrections, this approach risks Type I error inflation and yields lower statistical power than model-embedded simple effects analyses. We recommend model-based contrast methods for more robust inferences in future investigations. Another issue to be noted is that the study participants were young males, and the sample source was relatively homogeneous. Therefore, the generalizability of the research findings remains limited and cannot be directly extended to females, different age groups or individuals with musculoskeletal disorders. Lastly, this study did not incorporate concurrent electromyography, ultrasound elastography, or other imaging modalities, nor did it directly compare different dosage parameters. As such, the precise mechanisms underlying the stiffness-reducing effects of rESWT and the optimal parameter selection still require further investigation in subsequent studies.

## 6 Conclusion

This study found that rESWT may yield immediate effects in young males with hamstring tightness. It could reduce muscle stiffness in the mid-to-lower hamstring regions within a 5-minute short-term and improve hamstring flexibility, as reflected by SRT, AKE, and ASLR. Additionally, although there were no significant between-group differences in post-intervention raw SRT scores, the rESWT group showed substantially greater improvements in change magnitude than the control group. These findings tentatively suggest that rESWT has potential benefits for relieving hamstring tightness, modulating muscle mechanical properties, and enhancing posterior lower-extremity chain flexibility. Limited by the immediate and 5-minute short-term observation period and single sample source, issues such as the long-term efficacy and optimal intervention parameters of rESWT still require further validation through large-sample, long-term follow-up, multi-parameter controlled randomized studies. In future research, we will conduct long-term efficacy follow-ups, expand the sample sources, and combine other assessment methods (such as shear wave elastography or electromyography) to provide more comprehensive objective evidence for the effect of rESWT on improving hamstring tightness.

## Data Availability

The original contributions presented in the study are included in the article/**Supplementary Material**. Further inquiries can be directed to the corresponding authors.

## Supplementary material

Supplementary Material S1 Results of the nonparametric mixed-design ANOVA test and *post-hoc* analyses for ST muscle stiffness.

Supplementary Material S2 Nonparametric mixed-design ANOVA test results for the stiffness of the BF muscle.
