# Supplementary material for: Radial extracorporeal shock wave therapy modulates hamstring stiffness and flexibility in young males with hamstring tightness: a randomized controlled trial
Source: Front Physiol. 2026 Sep 7;17:1927662. doi: 10.3389/fphys.2026.1927662 (PMC13590330; doi:10.3389/fphys.2026.1927662)
Supplement: Supplementary file 1 [file Supplementaryfile1.docx]

Supplementary Material 1

Radial Extracorporeal Shock Wave Therapy Modulates Hamstring Stiffness and Flexibility in Young Males with Hamstring Tightness: A Randomized Controlled Trial

Meixia Zou^1^, Siqi Huang^1^, Kaiyao Hou^2^, Yiming Chen^1^, Peifeng Shen^1^, Wenjie Guo^1^, Yinglan Ye^1^, Hairong Tao^1^, Guotong Dong^1^, Man Hao^1^, Zhijie Zhang^3*^, Chunlong Liu^1*^

^1^Clinical Medical College of Acupuncture Moxibustion and Rehabilitation, Guangzhou University of Chinese Medicine, Guangzhou, China

^2^The First Clinical Medical School, Guangzhou University of Chinese Medicine, Guangzhou, China

^3^Orthopedic Hospital of Henan Province, Luoyang, China

*** Correspondence:**
Chunlong Liu, lcl05mpt@gzucm.edu.cn
Zhijie Zhang, [sportspt@163.com](mailto:sportspt@163.com)

**Supplementary Tables**

**Table S1** Nonparametric mixed-design ANOVA test results for the stiffness of the ST muscle.

| **Muscle** | **Effect** | | ***F*** | ***P_FDR_*** | ***η²*** |
| --- | --- | --- | --- | --- | --- |
| ST-25% | Main | Group | 0.239 | 0.626 | 0.002 |
|  |  | Time | 115.519 | <0.001* | 0.472 |
|  | Interaction | | 79.450 | <0.001* | 0.381 |
| ST-50% | Main | Group | 6.529 | 0.015* | 0.048 |
|  |  | Time | 120.133 | <0.001* | 0.482 |
|  | Interaction | | 68.133 | <0.001* | 0.346 |
| ST-75% | Main | Group | 2.119 | 0.166 | 0.016 |
|  |  | Time | 94.103 | <0.001* | 0.422 |
|  | Interaction | | 60.377 | <0.001* | 0.319 |

ST: semitendinosus; 25%, 50%, 75%: measured at 25%, 50%, 75% from the ischial tuberosity to the medial femoral epicondyles. * *P_FDR_* < 0.05. Effect size criteria: small effect *η²* = 0.01; medium effect *η²* = 0.06; large effect *η²* = 0.14.

**Table S2** Post-hoc within-group comparisons using the Friedman test for the stiffness of the ST muscle.

| **Site** | **Group** | ***χ^2^*** | ***P_FDR_*** |
| --- | --- | --- | --- |
| ST-25% | rESWT group | 95.176 | <0.001* |
|  | Control group | 29.566 | <0.001* |
| ST-50% | rESWT group | 97.331 | <0.001* |
|  | Control group | 44.116 | <0.001* |
| ST-75% | rESWT group | 83.336 | <0.001* |
|  | Control group | 20.901 | <0.001* |

ST: semitendinosus; 25%, 50%, 75%: measured at 25%, 50%, 75% from the ischial tuberosity to the medial femoral epicondyles. * *P_FDR_* < 0.05.

**Table S3.** Post-hoc pairwise comparisons across time points within groups using Dunn’s test for the stiffness of the ST muscle.

| **Site** | **Group** | **Paired** | ***Z*** | ***P_FDR_*** | **Cliff’s *δ* [95%CI]** |
| --- | --- | --- | --- | --- | --- |
| ST-25% | rESWT group | T1 vs. T0 | 9.698 | <0.001* | -0.939 [-1.000, -0.856] |
|  |  | T2 vs. T0 | 5.766 | <0.001* | -0.848 [-0.977, -0.720] |
|  |  | T2 vs. T1 | -3.932 | <0.001* | 0.636 [0.449, 0.824] |
|  | Control group | T1 vs. T0 | 5.109 | <0.001* | -0.600 [-0.796, -0.404] |
|  |  | T2 vs. T0 | 0.941 | 0.347 | -0.092 [-0.336, 0.152] |
|  |  | T2 vs. T1 | -4.167 | <0.001* | 0.461 [0.244, 0.679] |
| ST-50% | rESWT group | T1 vs. T0 | 9.462 | <0.001* | -0.939 [-1.000, -0.856] |
|  |  | T2 vs. T0 | 7.151 | <0.001* | -0.985 [-1.000, -0.943] |
|  |  | T2 vs. T1 | -2.311 | 0.024* | 0.424 [0.204, 0.644] |
|  | Control group | T1 vs. T0 | 6.207 | <0.001* | -0.615 [-0.809, -0.422] |
|  |  | T2 vs. T0 | 1.057 | 0.314 | -0.231 [-0.469, 0.008] |
|  |  | T2 vs. T1 | -5.151 | <0.001* | 0.708 [0.535, 0.881] |
| ST-75% | rESWT group | T1 vs. T0 | 8.781 | <0.001* | -0.864 [-0.986, -0.741] |
|  |  | T2 vs. T0 | 6.551 | <0.001* | -0.909 [-1.000, -0.808] |
|  |  | T2 vs. T1 | -2.228 | 0.029* | 0.409 [0.187, 0.631] |
|  | Control group | T1 vs. T0 | 4.357 | <0.001* | -0.492 [-0.706, -0.279] |
|  |  | T2 vs. T0 | 0.978 | 0.353 | -0.123 [-0.366, 0.120] |
|  |  | T2 vs. T1 | -3.379 | 0.001* | 0.400 [0.175, 0.625] |

ST: semitendinosus; 25%, 50%, 75%: measured at 25%, 50%, 75% from the ischial tuberosity to the medial femoral epicondyles. T0: pre-intervention; T1: post-immediate intervention; T1: post-5 min intervention. * *P_FDR_* < 0.05. Effect size criteria: small effect |δ| = 0.147-0.330; medium effect |δ| = 0.330-0.474; large effect |δ| > 0.474. Effects with 95% CI excluding zero were considered statistically significant.

**Table S4.** Post-hoc between-group comparisons at each time point using Wilcoxon rank-sum tests for the stiffness of the ST muscle.

| **Site** | **Time** | ***Z*** | ***P_FDR_*** | **Cliff’s *δ* [95%CI]** |
| --- | --- | --- | --- | --- |
| ST-25% | pre | -1.093 | 0.367 | 0.111 [-0.061, 0.282] |
|  | post-immediate | -1.620 | 0.189 | -0.164 [-0.335, 0.006] |
|  | post-5min | -1.068 | 0.367 | -0.108 [-0.280, 0.063] |
| ST-50% | pre | -0.364 | 0.716 | 0.037 [-0.135, 0.210] |
|  | post-immediate | -4.185 | <0.001* | -0.424 [-0.580, -0.268] |
|  | post-5min | -3.998 | <0.001* | -0.405 [-0.563, -0.247] |
| ST-75% | pre | -0.859 | 0.439 | 0.087 [-0.085, 0.259] |
|  | post-immediate | -2.465 | 0.031* | -0.250 [-0.417, -0.082] |
|  | post-5min | -2.550 | 0.031* | -0.259 [-0.425, -0.091] |

ST: semitendinosus; 25%, 50%, 75%: measured at 25%, 50%, 75% from the ischial tuberosity to the medial femoral epicondyles. * *P_FDR_* < 0.05. Effect size criteria: small effect |δ| = 0.147-0.330; medium effect |δ| = 0.330-0.474; large effect |δ| > 0.474. Effects with 95% CI excluding zero were considered statistically significant.
