# Supplementary material for: Radial extracorporeal shock wave therapy modulates hamstring stiffness and flexibility in young males with hamstring tightness: a randomized controlled trial
Source: Front Physiol. 2026 Sep 7;17:1927662. doi: 10.3389/fphys.2026.1927662 (PMC13590330; doi:10.3389/fphys.2026.1927662)
Supplement: Supplementary file 2 [file Supplementaryfile2.docx]

Supplementary Material 2

Radial Extracorporeal Shock Wave Therapy Modulates Hamstring Stiffness and Flexibility in Young Males with Hamstring Tightness: A Randomized Controlled Trial

Meixia Zou^1^, Siqi Huang^1^, Kaiyao Hou^2^, Yiming Chen^1^, Peifeng Shen^1^, Wenjie Guo^1^, Yinglan Ye^1^, Hairong Tao^1^, Guotong Dong^1^, Man Hao^1^, Zhijie Zhang^3*^, Chunlong Liu^1*^

^1^Clinical Medical College of Acupuncture Moxibustion and Rehabilitation, Guangzhou University of Chinese Medicine, Guangzhou, China

^2^The First Clinical Medical School, Guangzhou University of Chinese Medicine, Guangzhou, China

^3^Orthopedic Hospital of Henan Province, Luoyang, China

*** Correspondence:**
Chunlong Liu, lcl05mpt@gzucm.edu.cn
Zhijie Zhang, [sportspt@163.com](mailto:sportspt@163.com)

**Supplementary Tables**

**Table S1** Nonparametric mixed-design ANOVA test results for the stiffness of the BF muscle.

| **Muscle** | **Effect** | | ***F*** | ***P_FDR_*** | ***η²*** |
| --- | --- | --- | --- | --- | --- |
| BF-25% | Main | Group | 0.742 | 0.391 | 0.006 |
|  |  | Time | 93.139 | <0.001* | 0.419 |
|  | Interaction | | 89.565 | <0.001* | 0.410 |
| BF-50% | Main | Group | 1.536 | 0.245 | 0.012 |
|  |  | Time | 103.399 | <0.001* | 0.445 |
|  | Interaction | | 52.713 | <0.001* | 0.290 |
| BF-75% | Main | Group | 3.636 | 0.076 | 0.027 |
|  |  | Time | 50.583 | <0.001* | 0.282 |
|  | Interaction | | 32.270 | <0.001* | 0.200 |

BF: biceps femoris; 25%, 50%, 75%: measured at 25%, 50%, 75% from the ischial tuberosity to lateral the femoral epicondyles. * *P_FDR_* < 0.05. Effect size criteria: small effect *η²* = 0.01; medium effect *η²* = 0.06; large effect *η²* = 0.14.

**Table S2** Post-hoc within-group comparisons using the Friedman test for the stiffness of the BF muscle.

| **Site** | **Group** | ***χ^2^*** | ***P_FDR_*** |
| --- | --- | --- | --- |
| BF-25% | rESWT group | 89.576 | <0.001* |
|  | Control group | 19.129 | <0.001* |
| BF-50% | rESWT group | 90.411 | <0.001* |
|  | Control group | 21.953 | <0.001* |
| BF-75% | rESWT group | 69.252 | <0.001* |
|  | Control group | 14.395 | 0.001* |

BF: biceps femoris; 25%, 50%, 75%: measured at 25%, 50%, 75% from the ischial tuberosity to the lateral femoral epicondyles. * *P_FDR_* < 0.05.

**Table S3.** Post-hoc pairwise comparisons across time points within groups using Dunn’s test for the stiffness of the BF muscle.

| **Site** | **Group** | **Paired** | ***Z*** | ***P_FDR_*** | **Cliff’s *δ* [95%CI]** |
| --- | --- | --- | --- | --- | --- |
| BF-25% | rESWT group | T1 vs. T0 | 9.226 | <0.001* | -0.939 [-1.000, -0.856] |
|  |  | T2 vs. T0 | 6.441 | <0.001* | -0.878 [-0.995, -0.763] |
|  |  | T2 vs. T1 | -2.785 | 0.006* | 0.455 [0.238, 0.671] |
|  | Control group | T1 vs. T0 | 3.852 | <0.001* | -0.353 [-0.583, -0.125] |
|  |  | T2 vs. T0 | 0.133 | 0.894 | -0.108 [-0.351, 0.136] |
|  |  | T2 vs. T1 | -3.720 | <0.001* | 0.523 [0.314, 0.732] |
| BF-50% | rESWT group | T1 vs. T0 | 9.287 | <0.001* | -0.909 [-1.000, -0.808] |
|  |  | T2 vs. T0 | 6.410 | <0.001* | -0.909 [-1.000, -0.808] |
|  |  | T2 vs. T1 | -2.878 | 0.005* | 0.500 [0.289, 0.710] |
|  | Control group | T1 vs. T0 | 4.279 | <0.001* | -0.415 [-0.638, -0.193] |
|  |  | T2 vs. T0 | 0.485 | 0.678 | -0.138 [-0.381, 0.104] |
|  |  | T2 vs. T1 | -3.793 | <0.001* | 0.523 [0.314, 0.732] |
| BF-75% | rESWT group | T1 vs. T0 | 7.863 | <0.001* | -0.848 [-0.977, -0.720] |
|  |  | T2 vs. T0 | 6.291 | <0.001* | -0.788 [-0.928, -0.638] |
|  |  | T2 vs. T1 | -1.573 | 0.130 | 0.242 [0.007, 0.478] |
|  | Control group | T1 vs. T0 | 3.067 | 0.002* | -0.323 [-0.555, -0.091] |
|  |  | T2 vs. T0 | -0.400 | 0.716 | 0.015 [-0.230, 0.260] |
|  |  | T2 vs. T1 | -3.468 | 0.001* | 0.431 [0.210, 0.652] |

BF: biceps femoris; 25%, 50%, 75%: measured at 25%, 50%, 75% from the ischial tuberosity to the lateral femoral epicondyles. T0: pre-intervention; T1: post-immediate intervention; T1: post-5 min intervention. * *P_FDR_* < 0.05. Effect size criteria: small effect |δ| = 0.147-0.330; medium effect |δ| = 0.330-0.474; large effect |δ| > 0.474. Effects with 95% CI excluding zero were considered statistically significant.

**Table S4.** Post-hoc between-group comparisons at each time point using Wilcoxon rank-sum tests for the stiffness of the BF muscle.

| **Site** | **Time** | ***Z*** | ***P_FDR_*** | **Cliff’s *δ* [95%CI]** |
| --- | --- | --- | --- | --- |
| BF-25% | pre | -0.725 | 0.527 | 0.074 [-0.098, 0.246] |
|  | post-immediate | -1.809 | 0.127 | -0.183 [-0.353, -0.013] |
|  | post-5min | -1.607 | 0.162 | -0.163 [-0.333, 0.007] |
| BF-50% | pre | -1.040 | 0.383 | 0.106 [-0.066, 0.277] |
|  | post-immediate | -2.334 | 0.059 | -0.237 [-0.404, -0.069] |
|  | post-5min | -2.200 | 0.062 | -0.223 [-0.391, -0.055] |
| BF-75% | pre | -0.012 | 0.991 | 0.001 [-0.171, 0.174] |
|  | post-immediate | -2.730 | 0.029* | -0.277 [-0.443, -0.111] |
|  | post-5min | -2.856 | 0.029* | -0.290 [-0.455, -0.124] |

BF: biceps femoris; 25%, 50%, 75%: measured at 25%, 50%, 75% from the ischial tuberosity to the lateral femoral epicondyles. * *P_FDR_* < 0.05. Effect size criteria: small effect |δ| = 0.147-0.330; medium effect |δ| = 0.330-0.474; large effect |δ| > 0.474. Effects with 95% CI excluding zero were considered statistically significant.
